# Oral Health among Adult Residents in Vilnius, Lithuania

**DOI:** 10.3390/ijerph19010582

**Published:** 2022-01-05

**Authors:** Milda Vitosyte, Alina Puriene, Indre Stankeviciene, Arunas Rimkevicius, Rita Trumpaite-Vanagiene, Jolanta Aleksejuniene, Lina Stangvaltaite-Mouhat

**Affiliations:** 1 Institute of Odontology, Faculty of Medicine, Vilnius University, LT-08217 Vilnius, Lithuania; m.vitosyte@gmail.com (M.V.); alina.puriene@mf.vu.lt (A.P.); indre.stankeviciene@mf.vu.lt (I.S.); arunas.rimkevicius@mf.vu.lt (A.R.); rita.trumpaite-vanagiene@mf.vu.lt (R.T.-V.); 2 Department of Oral Health Sciences, Faculty of Dentistry, The University of British Columbia, Vancouver, BC V6T 1Z3, Canada; jolanta@dentistry.ubc.ca; 3 Oral Health Centre of Expertise in Eastern Norway, 0369 Oslo, Norway; 4 Department of Clinical Dentistry, Faculty of Health Sciences, UiT The Arctic University of Norway, 9037 Tromso, Norway

**Keywords:** oral health, dental caries, periodontitis, oral examination, missing teeth

## Abstract

According to the World Health Organization (WHO) oral conditions may be determined by social, biological, behavioral, and psychosocial factors. The study assessed oral health status and its determinants associated with oral health conditions among adult residents in Vilnius, Lithuania. A total of 453 of 35–74-year-olds participated (response rate 63%). A self-reported questionnaire was administered. Dental caries experience (D_3_MFS score), periodontal probing depth (PPD), andnumber of missing teeth were assessed clinically. Data were analyzed using χ^2^ test, independent samples *t*-test, and multivariable linear regression. The mean (sd) of D_3_MFS scores was 67.3 (33.5), the mean (sd) number of teeth with PPD 4+ mm was 5.9 (5.3), prevalence of periodontitis was 33%, the mean (sd) number of missing teeth was 6.9 (6.8), and prevalence of total edentulism was 3.8%. Medication use was associated with all oral health conditions, while age was associated with caries experience, and missing teeth. Sugar-containing diet was associated with caries experience, and missing teeth, and smoking with caries experience and periodontal status. Systemic diseases were associated with periodontal status, while behavioral determinants, last dental visit, and use of fluoridated toothpaste were associated with missing teeth. Oral health status among adult Vilnius residents was poor. Oral conditions were associated with both biological and behavioral determinants. Oral health promotion should focus on modifying behavioral determinants.

## 1. Introduction

Oral health is an important component of general health, and a set of risk factors are common for both [1,2]. Oral diseases were recognized as a major public health problem as indicated by the 2017 Global Burden of Diseases study reporting that oral diseases impact 3.5 billion people worldwide [3]. Untreated dental caries and severe periodontitis are also among the ten most prevalent non-communicable diseases globally. Major oral illnesses can be avoided [4,5].

The etiology of oral conditions is complex and multifactorial. According to the World Health Organization (WHO) Social Determinants of Health Framework, oral conditions may be determined by social, biological, behavioral, and psychosocial factors [2]. Even though, this framework demonstrates that behavioral and psychosocial factors are only intermediate determinants of health, as these factors are shaped by one’s social environment. However, behavior modifications could also be promoted as a public health strategy [5]. Since the 20th century, the importance of disease prevention has expanded in many nations, and much experience has been gathered through community initiatives, population-based preventive activities, and individual preventive care [4,5].

Oral conditions have been the most prevalent non-communicable health problems in Lithuania [6]. The previous 1997/1998 Lithuanian National Oral Health Survey (LNOHS) found that Lithuania had one of the highest prevalence of caries, periodontal diseases, and missing teeth in Europe [7]. During the last few decades, the country experienced substantial economic and other changes while transitioning into a Western economy. Vilnius is an economic and cultural center of Lithuania, consisting of five major ethnicities, thus the current study focused on assessing oral health status and its determinants among adult residents in Vilnius. Compared to other European capital cities, Vilnius is a growing capital city with a steadily expanding population. It has been observed that in the past 20 years residents of Lithuania’s rural districts have declared their domicile in the capital. In addition, emigrants coming back to Lithuania also usually establish in the capital city rather than their birthplace. However, up to now, there has not been any study conducted on the oral health status of Vilnius residents.

The current study assessed oral health status and tested several determinants based on the WHO Social Determinants of Health Framework (social, biological, behavioral, and psychosocial) in association with oral health among adults in Vilnius capital city.

## 2. Materials and Methods

The current cross-sectional epidemiological study used data collected during the 2017–2019 Lithuanian National Oral Health Survey. A stratified random sampling selected 35–74-year-old Lithuanians from the five largest cities and one randomly selected periurban/rural area from each of the 10 counties. Calculations for the necessary sample size showed that we needed to recruit a minimum of 300 participants from each pre-selected age group: 35–44, 45–54, 55–64, and 65–74 years old. The calculated sample size was multiplied by 1.5 to adjust for the study design and further increased due to an expected 50% non-participation rate based on the previous 1997/1998 national Lithuanian study [8,9]. This study uses data collected only in Vilnius city. A total of 720 patients were invited in Vilnius, of which 453 agreed to participate (response rate 63.0%) and signed a written informed consent.

### 2.1. Clinical Examinations

One trained and calibrated examiner (IS) assisted by a dental assistant performed all clinical examinations. The clinical assessment was based on 28 teeth, where adults were seated in a dental chair and examined under the dental unit lamp and a compressed air flow. For clinical examinations we used a plane mouth mirror and a CPITN periodontal probe, which is recommended by the WHO [10]. The following WHO indices were recorded as study outcomes: dental caries experience indicated by the total numbers of decayed (D_3_), missing (M), and filled (F) surfaces (D_3_MFS score), the total number of teeth with periodontal probing depth PPD 4+ mm, and the number of missing teeth [10]. The intra- and inter-examiner reliabilities were calculated using duplicate recordings of 10 randomly selected patients, which were not included in the main study. The intra-class correlation coefficients for the total numbers of teeth with PPD 4+ mm, decayed (D_3_), missing (M), and filled (F) surfaces were 0.95. 1.00, 0.99, and 1.00, respectively. These levels of intra-examiner agreement were considered satisfactory.

### 2.2. Questionnaire

The participants completed the WHO Oral Health Questionnaire for Adults [10]. An identification code was given to each of the questionnaires, matching the clinical examination form. The principal investigator was responsible for safeguarding the list with codes related to personal information of attending participants. Only coded depersonalized data were used by other investigators participating in the study.

The English version was translated into Lithuanian, Russian and Polish languages and then back into the English language. In addition, three questions were added concerning: the presence of self-reported systemic diseases and their specification, the use of medications and their specification, and participants’ ethnicity. The Perceived Stress Scale-10 (PSS-10) measured participants’ psychological distress. The questionnaire was a pilot tested on 10 adults who were not part of the main study, subsequently the questionnaire was revised, where needed. The internal consistency of the PSS-10 scale was acceptable as indicated by the Cronbach’s alpha of 0.75.

The operationalization of original study variables and their categorization for analyses are presented in Table 1. The variables were selected based on the WHO Social Determinants of Health framework [2]. Social determinants included information about sex, ethnicity, and education, biological determinants included age, presence of systemic diseases, and use of medication, behavioral determinants were smoking frequency, alcohol use, sugar-containing diet, time of the last dental visit, use of fluoridated toothpaste, and toothbrushing frequency. Psychosocial determinants were measured by the PSS-10.

### 2.3. Statistical Analyses

The SPSS software version 28.0 (IBM, Somers, NY, USA) was used for statistical analyses. Data analyses included descriptive statistics, the χ^2^ test and independent samples *t*-test to identify sex-related differences. Multivariable linear regression analyses were employed to identify the significant determinants of the following oral conditions (study outcomes): dental caries experience (D_3_MFS scores), periodontal status (ratio of PPD 4+ mm), and the number of missing teeth. All regression models were significant (*p* < 0.001). The hierarchical regression (blockwise method) was used, where social determinants (sex, ethnicity, education) were entered in the first block, biological determinants (age, medication use, and systemic diseases) in the second block, behavioral determinants (smoking, alcohol use, tooth brushing frequency, use of fluoridated toothpaste, last dental visit, and sugar-sweetened diet) in the third block and psychosocial determinants (perceived stress) in the fourth block. To acquire robust confidence intervals for the model parameters, which did not rely on normality assumption, the Bootstrap option was used. The level of significance was set at *p* < 0.050. Models’ predictors were presented with B coefficients and 95% bias corrected accelerated confidence intervals (BCa 95%CIs). The assumption of no multicollinearity as indicated by high tolerance and low VIF values was fulfilled in all models. To determine how much variance in outcomes each group of determinants can explain, the Adjusted R^2^ (Adj. R^2^) was recorded for the whole model and separately for each block.

## 3. Results

### 3.1. Socio-Demographic and Lifestyle Characteristics

The study sample consisted of 54.7% females (*n* = 258). Three females and two males opted not to complete the questionnaire, consequently they were excluded from the further investigation. Among females, the mean age (sd) was 54.7 (13.7) years and among males 52.4 (13.7) years. A significantly higher proportion of females (41.4%) than of males (25.6%) reported medication use and having systemic diseases (51.9% vs. 31.8%) and used fluoridated toothpaste (53.6% vs. 43.4%). There was a significant sex-related difference in sugar-containing diet consumption frequency between sexes (Table 2).

### 3.2. Outcome: Dental Caries Experience

Overall dental caries experience as indicated by the mean (sd) D_3_MFS score was 67.3 (33.5) surfaces with the median of 62.0 (interquartile range, IQR 128) surfaces. According to multivariable linear regression models (Table 3), the following significant determinants of higher D_3_MFS scores were: age (B = 0.48 BCa 95%CI 0.22–0.73), medication use (B = 14.79, BCa 95%CI 7.54; 22.25), smoking (B = 9.58, BCa 95%CI 3.44–14.47), sugar-containing diet-moderate vs. lower (B = 8.21, BCa 95%CI 0.66–14.89); and higher vs. lower (B = 15.84, BCa 95%CI 7.21–24.30).

### 3.3. Outcome: Periodontal Status

The mean (sd) number of teeth with PPD 4+ mm was 5.9 (5.3) with a median being 5.0 teeth (IQR 24). The prevalence of periodontitis as indicated by having a higher % ratio of PPD 4+ mm was 33%. According to multivariable linear regression analysis (Table 3), the significant predictors of periodontal status were medication use (B = 1.53, BCa 95%CI 0.52–2.54), systemic diseases (B = 1.89, 0.16–3.56), and smoking frequency (B = 2.37, BCa 95%CI 1.49–3.32).

### 3.4. Outcome: Numbers of Missing Teeth

The mean (sd) number of missing teeth was 6.9 (6.8) with a median being 5.0 (IQR 28) teeth. The prevalence of total edentulism was 3.8%. According to multivariable linear regression analysis (Table 3), age (B = 0.08, BCa 95%CI 0.29–0.14), medication use (B = 1.58, BCa 95%CI 0.19–2.86), sugar-containing diet-higher vs. lower (B = 2.78, BCa 95%CI 0.99–4.57), timeof the last dental visit (B = 2.31, BCa 95%CI 0.93–4.03) and use of fluoridated toothpaste (B = 1.83, BCa 95%CI 0.72–2.87) were associated with higher numbers of missing teeth (Table 3).

## 4. Discussion

The current cross-sectional epidemiological study assessed oral health status and its determinants among adult residents of Vilnius, Lithuania. Our response rate was 63%, consequently potential self-selection bias could not be ruled out. Due to the cross-sectional study design, we were unable to infer any causality between determinants and three oral health outcomes. In this study, the statistically significant associations had wide confidence intervals, which is a common finding for chronic diseases having a multifactorial etiology. However, it may suggest that the results should be interpreted with caution. Self-reported questionnaires were used to gather information about determinants, this also might have introduced some information bias. On the other hand, objective clinical data collection following the WHO guidelines increases both internal and external validity. In 2021, there were 54% permanent female residents in Vilnius and in this study female participants constituted 57% of all participants, therefore our results could be generalized to all adult Vilnius citizens.

In the current study, dental caries experience, indicated by a mean (sd) D_3_MFS scoreof 67.3 (33.5), was higher compared to Denmark (aged 18–96) and Oslo, Norway (35-year-olds), where mean DMFS scores were 61.0 and 26.1, respectively [11,12]. The difference with Oslo city could be explained by having older participants in our study. Similarly, the prevalence of periodontitis (69.0%) was higher among Vilnius residents as compared to 18–75-year-olds from Finnmark county, Northern Norway (49.7%) [13]. Similarly, prevalence of edentulism among Vilnius residents (3.8%) was higher compared to 18+ year-olds in Jönköping, Sweden (1.0%), but lower than in Portugal (>15-year-olds) (6.4%) [14,15]. Therefore, the oral health status among adult citizens in Vilnius compared to available data from European countries and cities can be defined as poor.

In our study, one of the biological determinants, use of medications, was associated with all three oral health-related outcomes. Many medications, e.g., tricyclic antidepressants, antihistamines, and beta blockers reduce salivary flow resulting in mouth dryness, a known risk factor for oral conditions [16,17]. This finding illustrates a link between general and oral health. In our study, the majority of the participants using medications on a regular basis, reported using medicine against hypertension, this group also included use of beta blockers. In our study, the subsequent multivariable analyses to look for associations with the specific medications were not possible due to the relatively small size in subgroups.

Another biological determinant, older age, was a significant determinant of higher caries experience and of a higher number of missing teeth. The severity of destruction of oral tissues increases with age and this may be linked to a variety of different health conditions, particularly in middle or older age [18,19]. In addition, DMFS scores, measuring the total cumulative dental caries experience, are highly dependent on age.

The higher dental caries experience and higher numbers of missing teeth were associated with a higher intake of sugar-containing diet. Similar results were reported elsewhere indicating that a sugar-containing diet may be a major risk factor for dental caries [20,21,22]. Moreover, according to literature, once caries has developed, invasive treatment is the primary strategy for halting its progression, and failure to treat will almost certainly result in tooth loss [23]. One may deduct that the main reason for missing teeth as observed in our study is due to dental caries suggesting a sugar-containing diet as a pathway.

Smoking frequency was significantly associated with both dental caries experience and periodontal status. This finding is in line with a recent systematic review and meta-analysis, which concluded that there was a correlation between tobacco smoking and increased risk for dental caries [24]. Although several theories were suggested, the processes through which tobacco use influences the development of two main oral diseases, namely dental caries and periodontitis, are not well understood [24,25]. Smoking influences the composition of the microbiota, the immunological response, and the periodontal healing ability. Smoking is believed to increase adherence of *Streptococcus Mutans* that uses sucrose to support its metabolism, and its metabolites are primarily responsible for adhesion of microbiota and caries formation, altering the composition of the subgingival biofilms, resulting in periodontal infections [24]. Additionally, smoking has been linked to a delay in the recruitment and migration of neutrophils into periodontal tissues, consequently impairing the acute immune response. This would increase the threshold of aggressiveness required to begin the inflammatory cascade in periodontal tissues, potentially leading to a faster tooth loss [25].

In our study, periodontal status was associated with systemic diseases. In literature, diabetes, cardiovascular diseases, rheumatoid arthritis, HIV, chronic kidney, respiratory diseases, and neurologic conditions have been linked to periodontal diseases [26,27]. These findings support the notion that oral health is a part of general health sharing known and unknown common risk factors. The majority of our participants who had systemic diseases, reported cardiovascular diseases, diabetes and thyroid diseases. The subsequent analyses for the associations between specific systemic diseases and oral health conditions were not possible in this study due to a relatively small size in subgroups concerning the aforementioned systemic diseases.

Fluoridated toothpaste and time of the last dental visit were significantly associated with higher numbers of missing teeth. It is known that individuals not using fluoridated toothpaste have a higher chance of developing carious lesions, subsequently leading to the loss of teeth [28]. What was interesting was that in our study the use of fluoridated toothpaste was not associated with dental caries experience, contrary to sugar-sweetened diet. This finding may suggest that sugar-sweetened diet might be a more important predictor of dental caries compared to fluoridated toothpaste use in our study sample. Furthermore, it has been shown that individuals who had a recent dental visit, had lower odds for higher number of missing teeth [29,30]. These findings are in line with our study, which showed that participants who had a last dental visit more than a year ago, had more missing teeth. This highlights the importance of regular dental visits.

The poor oral health among adult citizens in Vilnius we observed and identified determines a call for action. However, up to date, there is no department responsible for dental public health in the Ministry of Health of the Republic of Lithuania. Consequently, this situation results in a lack of dental health policies. The provision of dental services is partly left to a private sector, posing major obstacles to the development of health promotion programs [5]. Therefore, the Vilnius City Municipality should place a greater emphasis on the importance of oral health and support its maintenance.

According to press reports, Vilnius is the fourth fastest growing city in Europe, leaving behind London, Rome, Stockholm, and Oslo. The city is expanding at the expense of suburbs. According to Eurostat statistics (2018), Lithuania ranks second among European Union (EU) Member States in terms of highest numbers of dentistry graduates in 2018 (6.4 graduates per 100,000 inhabitants and a total of 2758 practicing dentists), with a high dentist-to-population ratio (1.2 dentists per 1000 residents) when compared to other countries and the EU average. In 2014, there were 587 practicing dentists in the Vilnius region, indicating that the majority of dental practitioners work in the city with only 3.6% working in the Vilnius suburbs. Due to a shortage of dentists in the Vilnius suburbs, dentists in suburban areas may provide only emergency dental care with less emphasis placed on the prevention of oral diseases. Unsurprisingly, around one third of dentists in Vilnius city reported a patient shortage [31]. Of importance, this general oversupply of dentists in one area may result in patient overtreatment. We believe that modification in the distribution of dental offices may result in more effective and higher-quality oral health care.

In the present study, both biological and behavioral determinants were associated with oral health outcomes. The Common Risk Factor Approach (CRFA) has been introduced for a while and it demonstrates that oral and general health conditions share some common determinants [32]. This approach is relevant for non-communicable general and oral health conditions, mainly by focusing on modifiable behavioral risk factors such as reducing sugar intake and tobacco use. Several high-income countries took into consideration nutrition and reduced sugar intake towards the prevention of non-communicable oral and general health conditions. Official guidelines for limiting sugar consumption were implemented by raising taxes on sugar-containing products (e.g., Great Britain, Spain) [33,34]. This whole population-centered preventive strategy is consistent with the WHO sugar agenda, which also calls oral health practitioners to take action. Additionally, taxation may be imposed on tobacco and, as e-cigarettes gain popularity, on them as well [35]. According to studies, daily expenses for e-cigarettes are calculated to be even greater than for cigarettes, and e-cigarettes may be as harmful as tobacco [36]. It is advised to impose an excise tax on cigarettes using the CRFA approach. This approach can also be used in Lithuania, simultaneously having in mind the overcrowding of dental practitioners, a lack of focus on oral disease prevention, and the ties between general health, oral health, and common behavioral determinants, which were also illustrated in this report.

## 5. Conclusions

The current study demonstrates that the oral health status among adult Vilnius residents is poor. Oral conditions were associated with biological and behavioral determinants. Significant determinants of oral health conditions were age, medication use, systemic diseases, smoking frequency, higher intake of sugar-containing diet, time of the last dental visit, and use of fluoridated toothpaste. Therefore, oral health promotion and prevention strategies should focus on behavioral determinants common for oral and general health.

## Figures and Tables

**Table 1:** Operationalization of study variables and their categorization for statistical analyses.

Variables	Questions	Original Responses	Categorization for Analyses
Social determinants			
Sex	What is your sex?	Male Female	Male Female
Ethnicity	What is your ethnicity?	Lithuanian Russian Belorussian Ukrainian Polish Other	Lithuanian (1) Other (2–6)
Education	Years in school	In full years	In full years
Biological determinants			
Age	How old are you?	In full years	In full years
Systemic diseases	Do you have any systemtic diseases?	No Yes	No Yes
	What kind of systemtic diseases do you have?	Specifying the diseases	Cardiovascular diseases Diabetes Thyroid diseases Other diseases
Use of medication	Do you use any medications on a regular basis?	No Yes	No Yes
	What kind of medications do you use?	Specifying the medications	For cardiovascular diseases For diabetes For thyroid diseases Other medications
Behavioral determinants			
Smoking frequency (Cigarettes)	How often do you smoke cigarettes?	Never Seldom Several times a month Once a week Several times a week Everyday	Never (1) Smoker (2–6)
Alcohol use	During the past 30 days, on the days you drank alcohol, how many drinks did you usually drink per day?	<1 drink 1 drink 2 drinks 3 drinks 4 drinks 5 or more drinks Did not drink alcohol during past 30 days	Did not use alcohol (7) Used alcohol (1–6)
Sugar-containing diet	How often do you eat or drink any of the following foods, even in small quantities? (cake, sweet buns/breads, jam, honey, sweets, candies, soft drinks, tea with sugar, coffee with sugar)	Never/seldom Several times a month Once a week Several times a week Every day Several times a day	Lower intake of sweets/sugar beverages (sum score ≤ 16) Moderate intake of sweets/sugar beverages (sum score 17–27) Higher intake of sweets/sugar beverages (sum score ≥ 28) Used as a dummy variable with category 1 as reference.
Time of the last dental visit	How long is it since you last saw a dentist?	<6 months Within 6–12 months 1 year but <2 years ≥2 years but <5 years ≥5 years Never had dental visit	Last year (1–2) >1 year ago (3–6)
Use of fluoridated toothpaste	Do you use a toothpaste that contains fluoride?	Yes No Don’t know	Yes No/don’t know
Toothbrushing frequency	How often do you clean your teeth?	Never Once a month 2–3 times a month Once a week 2–6 times a week Once a day Twice or more a day	≥Twice a day (7) ≤Once a day (1–6)
Psychosocial determinants			
PSS-10	PSS-10 questions	Never Almost never Sometimes Quite often Very usually	Lower perceived stress (scores 0–13) Moderate perceived stress (scores 14–26) Higher perceived stress (scores 27–40) Used as a dummy variable with category 1 as reference.
Oral health outcomes (based on clinical examinations)			
Dental caries experience	Total number of decayed, missing, and filled surfaces (D_3_MFS score)	D_3_MFS score	D_3_MFS score
Periodontal status	Number of teeth with periodontal probing depth PPD 4+mm/number of teeth x 100%	Ratio of PPD 4+mm (%)	Ratio of PPD 4+mm (%)
Missing teeth	Total number of missing teeth	Number of missing teeth	Number of missing teeth

χ^2^ test for comparison of proportions between groups, and independent samples *t*-test for comparison of means between two groups.

**Table 2:** Sex-stratified social, biological, behavioral, and psychosocial determinants’ distribution among Vilnius residents.

Determinants	Females	Males	*p* Value
	258 (100%)	195 (100%)	
Social determinants			
Ethnicity			
Lithuanians	185 (71.7)	141 (72.3)	0.940
Other ethnic groups	72 (28.0)	54 (27.7)	
Education (in full years)	15.6 (3.1)	15.3 (3.2)	0.264
mean (sd)			
Biological determinants			
Age (in full years)			
mean (sd)	54.7 (13.7)	52.4 (13.7)	0.070
Systemic diseases			
No	124 (48.1)	133 (68.2)	0.014
Yes	134 (51.9)	62 (31.8)	
Specific systemtic diseases			
Cardiovascular diseases	42 (16.3)	20 (10.2)	
Diabetes	16 (6.2)	8 (4.1)	
Thyroid diseases	17 (6.6)	2 (1.0)	0.366
Other diseases	59 (22.9)	32 (16.4)	
Medication use			
No	151 (58.5)	145 (74.7)	0.034
Yes	107 (41.4)	50 (25.6)	
Specific medications			
For cardiovascular diseases	51 (19.8)	22 (11.3)	
For diabetes	9 (3.5)	4 (2.1)	
For thyroid diseases	17 (6.6)	2 (1.0)	0.525
Other medications	30 (11.6)	22 (11.3)	
Behavioral determinants			
Smoking frequency (cigarettes)			
Non-smoker	130 (50.4)	104 (53.3)	0.535
Smokers	128 (49.6)	91 (46.7)	
Alcohol use			
Does not use alcohol	137 (53.6)	96 (49.2)	0.368
Uses alcohol	119 (46.5)	99 (50.8)	
Sugar-containing diet			
Lower intake	77 (29.8)	40 (20.5)	0.013
Moderate intake	117 (45.3)	91 (46.7)	
Higher intake	64 (24.8)	64 (32.8)	
Time of the last dental visit			
Within last year	178 (69.0)	133 (68.2)	0.850
1 year or more ago	72 (28.8)	56 (28.7)	
Use of fluoridated toothpaste			
Yes	112 (43.4)	104 (53.6)	0.032
No/don’t know	146 (56.6)	90 (46.4)	
Toothbrushing frequency			
Twice a day or more	117 (46.8)	93 (48.9)	0.656
Once a day or less	133 (53.2)	97 (51.1)	
Psychosocial determinants			
Perceived stress			
Lower perceived stress	137 (54.2)	114 (59.4)	0.075
Medium perceived stress	73 (28.9)	59 (30.7)	
Higher perceived stress	43 (17.0)	19 (9.9)	
Oral health outcomes	mean (sd)	mean (sd)	
Dental caries experience	N = 257	N = 196	
D_3_MFS score (mean, sd)	67.6 (33.3)	66.9 (33.8)	0.834
Periodontal status	N = 249	N = 193	
Ratio of PPD 4+mm (%)	32.7 (28.8)	32.6 (32.3)	0.614
Number of missing teeth	N = 257	N = 196	
The mean number of missing teeth	6.9 (6.5)	6.8 (7.3)	0.767

χ^2^ test for comparison of proportions between groups, and independent samples *t*-test for comparison of means between two groups.

**Table 3:** Determinants of dental caries experience, periodontal status, and number of missing teeth in Vilnius residents (multivariable linear regression).

Study Outcomes									
	Dental Caries Experience			Periodontal Status			Number of Missing Teeth		
Determinants	*p* Value	B	BCa 95% CI	*p* Value	B	BCa 95% CI	*p* Value	B	BCa 95% CI
Social determinants									
Gender	0.359	−2.87	−8.75; 3.14	0.122	−0.84	−1.92; 0.12	0.846	−0.15	−1.50; 1.02
Ethnicity	0.529	2.06	−4.31; 8.74	0.754	−0.17	−1.24; 0.85	0.862	0.14	−1.34; 1.76
Education	0.974	−0.02	−1.44; 1.34	0.051	0.25	0.001; 0.51	0.122	0.23	−0.09; 0.52
Adj R^2^	−0.002			−0.004			−0.004		
Biological determinants									
Age	0.001	0.48	0.22; 0.73	0.068	0.04	−006; 0.09	0.003	0.08	.29; 0.14
Systemic diseases	0.665	−1.96	−11.07; 7.92	0.025	1.89	0.16; 3.56	0.950	0.05	−1.93; 2.07
Medication use	0.001	14.79	7.54; 22.25	0.007	1.53	0.52; 2.54	0.031	1.58	0.19; 2.86
Adj R^2^	0.149			0.065			0.065		
Behavioral determinants									
Smoking frequency	0.002	9.58	3.44; 14.47	0.001	2.37	1.49; 3.32	0.393	0.55	−0.69; 1.83
Alcohol use	0.113	−4.79	−10.36; 1.53	0.651	−0.23	−1.29; 0.87	0.767	−0.19	−1.35; 1.20
Sugar-containing diet									
Moderate vs. lower	0.024	8.21	0.66; 14.89	0.385	−0.56	−1.83; 0.63	0.097	1.11	−0.33; 2.44
Higher vs. lower	0.001	15.84	7.21; 24.20	0.223	−0.87	−2.36; 0.62	0.003	2.78	0.99; 4.57
Time of the last dental visit	0.140	4.56	−1.87; 10.77	0.510	0.39	−0.81; 1.49	0.005	2.31	0.93; 4.03
Use of fluoridated toothpaste	0.063	5.34	−0.60; 11.50	0.449	0.37	−0.61; 1.40	0.002	1.83	0.72; 2.87
Toothbrushing frequency	0.088	5.02	−0.43; 10.32	0.121	0.82	−0.19; 1.79	0.105	1.02	−0.27; 2.21
Adj R^2^	0.215			0.108			0.135		
Psychosocial determinant									
Perceived stress									
Medium vs. lower	0.064	−5.78	−11.81; 0.56	0.409	0.49	−0.75; 1.74	0.244	−0.83	−2.23; 0.63
Higher vs. lower	0.602	2.35	−6.09; 11.12	0.326	0.78	−0.79; 2.54	0.499	−0.67	−2.55; 1.19
Adj R^2^	0.220			0.107			0.135		

BCa CI—bias corrected accelerated confidence intervals.

## Data Availability

The data that support the findings of this study are available from the author, [AP], on request.
